# Treatment with somatostatin analogs induces differentially expressed let-7c-5p and mir-3137 in small intestine neuroendocrine tumors

**DOI:** 10.1186/s12885-019-5794-y

**Published:** 2019-06-13

**Authors:** Florian Bösch, Alexandr V. Bazhin, Sabine Heublein, Katharina Brüwer, Thomas Knösel, Florian P. Reiter, Christoph J. Auernhammer, Markus O. Guba, Christine Spitzweg, Jens Werner, Martin K. Angele

**Affiliations:** 1 Department of General, Visceral, and Transplant Surgery, Ludwig-Maximilians-University Munich, Marchioninistr. 15, 81377 Munich, Germany; 2 Department of Obstetrics and Gynecology, Heidelberg University Hospital, Heidelberg, Germany; 3 Institute of Pathology, Ludwig-Maximilians-University (LMU) Munich, Munich, Germany; 4 Department of Medicine II, University Hospital, LMU Munich, Munich, Germany; 5 Department of Internal Medicine 4, Ludwig-Maximilians-University Munich, Munich, Germany; 6 Interdisciplinary Center of Neuroendocrine Tumors of the GastroEnteroPancreatic System, Ludwig-Maximilians-University Munich, Munich, Germany

**Keywords:** Neuroendocrine tumor, microRNA, Somatostatin, Intra-individual

## Abstract

**Background:**

Distant metastases frequently occur in gastroenteropancreatic neuroendocrine tumors. If hepatic surgery is not feasible, patients are treated with somatostatin analogs. However, the underlying mechanisms of action of this treatment remain to be defined. The aim of the present study was to analyze the micro-RNA expression profile inter-individually before and after the treatment with somatostatin analogs.

**Material and methods:**

Tumor specimens of all included patients (*n* = 8) before and after the onset of a therapy with somatostatin analogs were analyzed and a micro-RNA expression profile (754 micro-RNAs) of each probe was generated. This analysis in an intra-individual setting was selected to avoid bias from inter-individual differences. The micro-RNA expression profiles were validated by qPCR. Patients with any other systemic treatment were excluded from the present study.

**Results:**

Eight patients were included in the present study of which all had neuroendocrine tumors of the small intestine with diffuse hepatic metastases. Grouped analyses revealed that 15 micro-RNAs were differentially expressed (3 up- and 12 downregulated) after the exposure to somatostatin analogs. Additionally, let-7c-5p and mir-3137 are concordantly regulated in the inter-individually analysis.

**Conclusions:**

This is the first study analyzing the individual micro-RNA expression profile before and after a therapy with somatostatin analogs. Data from this study reveal that somatostatin analogs may in part exert their beneficial effects through an alteration in the micro-RNA expression profile.

## Background

Neuroendocrine tumors of the small intestine (si-NETs) account for 45% of gastroenteropancreatic neuroendocrine tumors (GEP-NETs) [1, 2]. Si-NETs are usually small, but frequently lead to lymph node metastases associated with a desmoplastic reaction of the mesentery [3]. Moreover, although si-NETs are slow growing tumors they frequently show liver metastases at the time of initial diagnosis [4]. Thereby, even small tumors with a favorable grading (commonly G1 or G2), can result in a deteriorated prognosis due to distant metastases.

First line systemic treatment of well-differentiated GEP-NETs consists of a biotherapy with somatostatin analogs (SSAs) [5–7]. SSAs bind to somatostatin receptors (SSTRs), which are abundantly expressed on GEP-NETs [8]. Binding of SSAs leads to an activation of SSTRs and an induction of complex intracellular signaling pathways with subsequent alterations in cell function (exocrine ability, growth, viability) [9]. The anti-proliferative properties of SSAs have been validated in two multicenter prospective studies [10, 11].

Micro-RNAs (miRNAs), small non-coding RNA molecules (18–25 nucleotides), have been shown to modulate cell proliferation, differentiation, and apoptosis. The modulation takes place at post-transcriptional levels and their specific role in cancer can be either as an oncogene or as a tumor suppressor [12–14]. MiRNAs can be detected by a fully automated, high throughput procedure. Thereby, these small molecules might gain increased interest as diagnostic and/or prognostic markers. Indeed, previous studies revealed that miRNAs may act as prognostic biomarkers for different cancer types [15–17] or even as targets for tumor directed therapy [18]. Furthermore, the various entities of GEP-NETs (pancreas, small intestine, etc.) have each a distinct miRNA expression profile [19–21] and miRNAs might act as biomarkers [22, 23].

So far the exact underlying mechanisms of action of the SSA therapy need to be defined. We therefore addressed the present study to analyze individual miRNA expression profiles before and after SSA treatment.

## Material and methods

### Study cohort

Data of patients diagnosed with a GEP-NET who underwent surgery at our Department between 01/2000 and 12/2017 were collected in a prospective led database. For inclusion in the present study, a tumor specimen from every single patient before the onset of the biotherapy (group A) and a tumor sample after the onset of the biotherapy (group B) were necessary. Patients with any other systemic anti-tumor therapy were excluded. Eight patients met all criteria and were included in the present study (Table 1). Patients were selected from a surgical collective published earlier [3, 24] and were analyzed in this work in respect to the miRNA expression profile [25].

**Table 1:** Patients included in the present analysis (SSA: somatostatin analog)

Patient	Source before SSA	Source after SSA	Treatment period (months)
1	Liver metastasis	Primary tumor	3
2	Liver metastasis	Primary tumor	13
3	Liver metastasis	Primary tumor	5
4	Primary tumor	Liver metastasis	2
5	Liver metastasis	Primary tumor	1
6	Liver metastasis	Primary tumor	1
7	Liver metastasis	Primary tumor	1
8	Liver metastasis	Primary tumor	1

All patients underwent complete primary tumor resection at the Department of General, Visceral and Transplant Surgery at the Ludwig-Maximilians-University of Munich, Germany. Every specimen underwent routine processing and examination at the Institute of Pathology at the Ludwig-Maximilians-University of Munich, Germany.

#### RNA and miRNA isolation

Freshly sliced formalin-fixed paraffin-embedded (FFPE) tumor sections were used for the experiments which were done under sterile, RNAse, and DNAse free conditions. RNA extraction was done by microscope assisted microdissection supervised by an experienced pathologist (T.K.) with special expertise in GEP-NET pathology. Before microdissection the tumor area of each specimen was precisely marked on a hematoxylin and eosin stained serial slide to exclude necrotic area or adjacent tissue. The dissected tumor tissue was transferred into 1.5 ml tubes and further purified by using the miRNeasy FFPE Kit (Qiagen, Hilden) according manufacturer’s instructions. NanoDrop 2000 (Thermo Fisher Scientific, Waltham, MA) spectrophotometer was used to quantify the amount and the quality of nucleotide acids and only samples, which passed the quality control (A260/280 > 2.0, clear single RNA peak) were further processed.

#### Analysis of differentially expressed miRNAs

Pre-amplification of cDNA was performed with miRNA specific primers as provided with the Megaplex™ Primer Pools, Human Pools Set v3.0 (Applied Biosystems). Fluorescently-labeled miRNA were prepared according to Agilent protocol “miRNA Complete Labeling and Hyb Kit”. 100 ng labeled miRNA sample were hybridized for at least 20 h at 55 °C on Agilent human miRNA Microarray Release 21.0, 8x60k. The cDNAs were then diluted and loaded onto a TaqMan® Array Human MicroRNA A + B Cards Set (Applied Biosystems) and qRT-PCR run performed. RNU6-2_11 and RNU6_11 served as housekeeping genes on all Taqman Low Density Array (TLDA) cards run. Fold changes in expression were calculated for all miRNAs by dividing mean values from group B with group A. Gene Expression Microarrays were scanned using the Agilent Scanner G2505C. The scanned images were analyzed with Feature Extraction Software (Agilent technologies) using default settings.

#### Reverse transcription and qRT-PCR

miRNA was reverse-transcribed using the miScript II RT Kit (Qiagen) according to manufacturer’s protocol. The cDNA was then diluted with RNase-free water and frozen until qPCR. cDNA was thawed and qPCR was performed using the miScript SYBR Green PCR Kit (Qiagen) and the 10x miScript Primer Assays (Qiagen) for the miRNAs of interest according to manufacturer’s protocol as described elsewhere [26]. The runs were performed on a StepOne Plus real-time PCR system (Applied Biosystems). RNU6-2_11 served as housekeeping gene. Fold changes in expression were calculated for all miRNAs using the delta Ct (dCt) method (dCT (target sample) = CT (target gene) – CT (housekeeping gene)).

### Statistical analyses

All statistical analyses were performed by using Prism 6.0 for Mac (GraphPad Software, Inc., La Jolla, CA).

## Results

### Study cohort

In total, the database consists of 517 patients who underwent surgery for a GEP-NET. Out of these patients eight patients eligible for the study were identified. Every analyzed patient had a si-NET, lymph node and liver metastases. Mean age of the patients was 60.1 years (47.3–72.4 years) and five were female. Patient characteristics are displayed in Table 2.

**Table 2:** Patient characteristics of the eight analyzed patients (T: primary tumor stage; N1: lymph node metastases; M1: distant metastases; L0/1/x: lymphatic vessel infiltration (no/yes/no information); V0/1/x: Angioinvasion (no/yes/no information); Pn0/1/x: Perineural infiltration (no/yes/no information; unk.: unknown))

Patient	Age (years)	Primary tumor	Tumor staging	Size (cm)	Grading	Alive
1	66.8	small intestine	T3, N1 (50/82), M1, L1, V0, Pn1	1.3	2	yes
2	59.5	small intestine	T2, N1 (4/31), M1, L0, V0, Pn0	1.5	2	yes
3	57.0	small intestine	T4, N1 (8/28), M1, L1, V0, Pn1	1.6	2	yes
4	62.5	small intestine	T3, N1 (3/19), M1, L1, V1, Pn1	1.8	1	yes
5	47.3	small intestine	T3, N1 (5/24), M1, L1, V0, Pn0	1.5	2	yes
6	49.4	small intestine	T3, N1 (3/14), M1, L1, V0, Pn1	2.1	1	yes
7	65.9	small intestine	T4, N1 (3/16), M1, Lx, Vx, Pnx	unk.	2	no
8	72.4	small intestine	T3, N1 (5/50), M1, L1, V0, Pn0	0.8	1	yes

### MiRNA expression profile

**Fig. 1:**
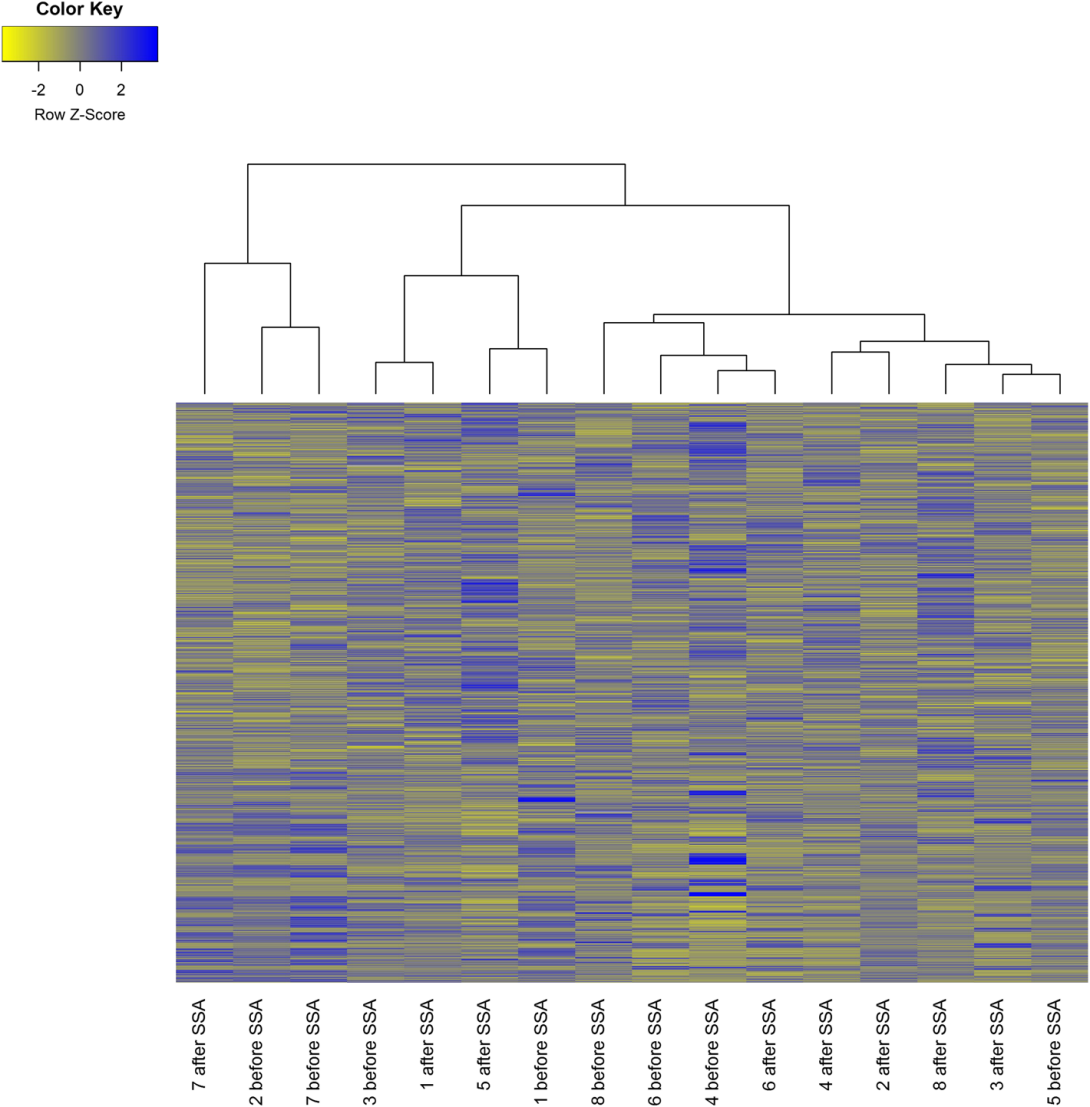
The heat map of the micro-RNA profiles analyzed

To attain an individual miRNA expression profile of every patient as a baseline, in the first step, a tumor sample of every patient was analyzed before the onset of a biotherapy with SSAs. In the second step, the effect of SSAs was analyzed. Therefore, tumor specimens of the same patients were analyzed again after the onset of the biotherapy. For every analysis of a specimen a single array card was used and 758 miRNAs (754 target miRNAs and 4 control RNAs) were quantified by TLDA. Paired specimens (i.e. 1A vs. 1B; 2A vs. 2B) were analyzed to clearly identify effects of a SSA treatment on the miRNA expression profile. By the use of this experimental design inter-individual alterations in the miRNA profile were ruled out. The analysis revealed that biotherapy with SSAs lead to an alteration in the miRNA expression profile (Fig. 1).

### Validation of miRNA by RT-PCR

Thirty-six miRNAs (20 most downregulated and 16 most upregulated, respectively), which were highly differentially expressed in TLDA cards, were identified by grouping (1A-8A vs. 1B-8B) the results. To validate the expression of these 36 miRNAs, qPCR of the same eight samples before and after treatment were analyzed. Although validation by qPCR was conducted for every single specimen the results were grouped thereafter.

The validation of the 36 miRNAs confirmed that 15 miRNAs are expressed differentially. SSA treatment induced an upregulation of three miRNAs (let-7c-5p, mir-24-3p, and mir-215-5p) and a downregulation of twelve miRNAs (mir-10a-3p, mir-185-3p, mir-339-5p, mir-371a-5p, mir-4436b-5p, mir-4653-3p, mir-4793-3p, mir-619-5p, mir-1226-3p, mir-3137, mir-4455, and mir-4656) (Table 3).

**Table 3:** Differentially expressed micro-RNAs, which were validated by PCR for the entire collective

microRNA	Fold change
let-7c-5p	9.53
mir-10a-3p	0.88
mir-24-3p	4.76
mir-185-3p	0.7
mir-215-5p	12.64
mir-339-5p	0.75
mir-371a-5p	0.36
mir-619-5p	0.75
mir-1226-3p	0.56
mir-3137	0.44
mir-4436b-5p	0.97
mir-4455	0.52
mir-4653-3p	0.7
mir-4656	0.73
mir-4793-3p	0.55

### MiRNA expression in single patients before and after SSA therapy

As mentioned above, the grouped miRNA expression profile is different before and after SSA therapy. In the next step, the differences in the miRNA expression profile for each patient had to be elucidated. Therefore, the individual qPCR values were compared.

**Fig. 2:**
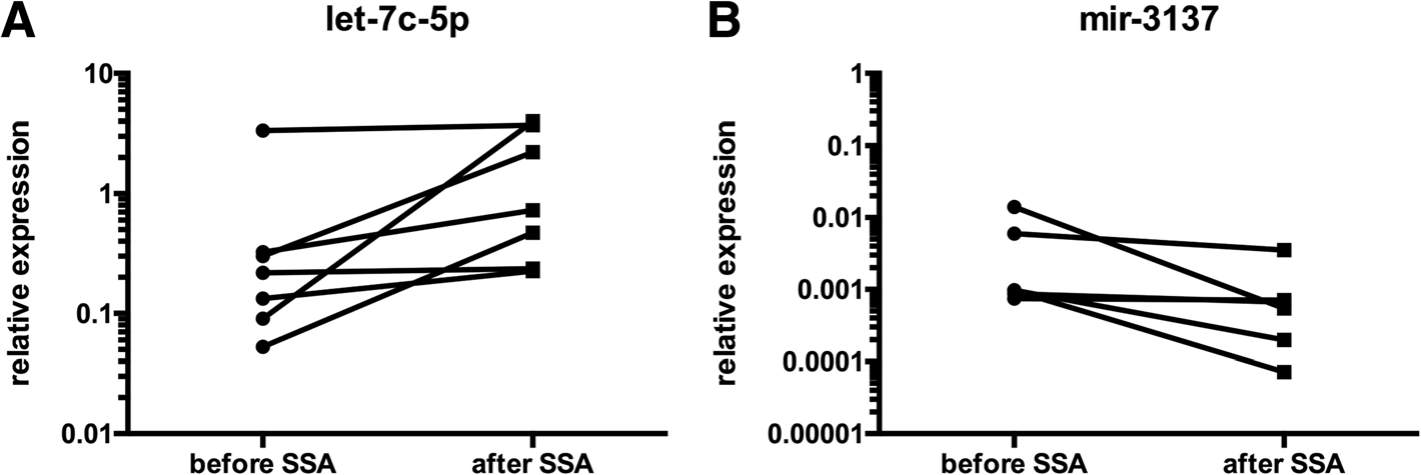
**a** Expression levels of the uniformly regulated micro-RNAs. let-7c-5p could be analyzed in patients 2–8 and was concordantly regulated. **b** mir-3137 could be analyzed in patients 2 and 4–8 and was concordantly regulated

This analysis revealed that let-7c-5p was concordantly upregulated in every patient after SSA therapy (Fig. 2a). Additionally, mir-3137 was concordantly downregulated in every patient after SSA therapy (Fig. 2b). The grouped analyses revealed that let-7c-5p was upregulated 9.53 times and mir-3137 was downregulated 0.44 times.

## Discussion

GEP-NETs demand more attention since the incidence is increasing [27]. The clinical importance of this entity is further underlined by the fact that even small tumors may lead to distant metastases [24]. The most validated prognostic marker is tumor grading, but si-NETs are commonly well-differentiated (G1 or G2). Thus, novel diagnostic biomarkers are needed. Moreover, there are no prognostic markers or tools to monitor therapeutic effects. Previous studies revealed an important role of miRNAs in various tumors that these can either act as an oncogene or a tumor suppressor. MiRNAs show a high stability in vitro and persistence in vivo. Therefore, analyzing miRNA signatures is even in FFPE tissue highly sensitive [28]. Furthermore, it was shown that the blockade or the replacement of a distinct miRNA can be used as a therapeutic approach [18]. Moreover, miRNAs seem to play a role in NETs and can be used as a biomarker, as demonstrated recently [22, 29, 30].

This is the first study analyzing the individual effect of a biotherapy with SSAs on the miRNA expression profile in si-NETs. The questioning of the present study cares aim to exclude inter-individual differences, subsequently only patients with tumor samples available before and after the onset of biotherapy were included. The generation of controls would lead to inter-individual differences and further hamper the interpretation of the results. In addition, miRNA expression profile alterations might be time dependent. But, the effects of time on the miRNA signature have not been investigated so far. In this respect, recent publications used normal tissue of the small intestine or the liver [30–32] as controls. But, this approach of generating a control collective was not appropriate for this analysis. Additionally, patients receiving any other systemic therapy were excluded in the present study. Thus, a very uniform study collective was generated and subsequently analyzed. It should be noted that the results of the present study analyzing si-NETs cannot be automatically extrapolated for NETs outside the small intestine. As a result of this approach the direct effect of a SSA therapy could be evaluated on the post-transcriptional level. MiRNAs will gain further attention since they can be detected fully automated in a high throughput technique. One would speculate that the protein expression might be different as a consequence of biotherapy. However, these effects have to be validated in a first step with in vitro trials since miRNAs have an abundance of target genes. Furthermore, the patient collective might be too small to demonstrate clinical effects. Nonetheless, the present study provides miRNA profiles to stimulate further research and evaluate the clinical relevance in a larger patient collective in a prospective multicenter study. Additionally, it is possible to determine miRNA profiles in serum samples, making liquid biopsies possible [29, 33, 34]. It was demonstrated in colorectal cancer that a distinct expression profile is associated with deteriorated survival rates [15]. In particular for example, mir-215 seems to be a suppressor of tumor progression in colorectal cancer [35, 36]. Additionally, it was seen in ovarian cancer, non-small cell lung cancer (NSCLC), and breast cancer that specific miRNAs lead to a shortened tumor-free survival [37–39]. In this respect, mir-215 might be a key player. In vitro studies demonstrated that the upregulation of mir-215 leads to apoptosis in colorectal and NSCLC cancer cells [36, 38]. In line with these findings, the present miRNA profiling revealed that after SSA treatment mir-215 was highly upregulated indicating a potential antitumoral mechanism of action of SSA-therapy. The results of the present study suggest that miRNAs represent a promising tool to monitor antitumor therapy. Moreover, future multicenter prospective studies should aim to clarify if biotherapy induces long-term alterations in miRNA expression profiles.

There are only few studies highlighting the effect of miRNAs and neuroendocrine tumors [19–21]. In this respect, the results of this analysis suggest that biotherapy directly influences the miRNA expression profile. The differences seen were intra-individual changes most probably induced by somatostatin therapy. There is a growing body of evidence, which suggests that miRNAs might serve as biomarkers in the near future. Panarelli et al. recently published their work on miRNA signatures and add further evidence for the importance of miRNAs. They have demonstrated that GEP-NETs can be classified and graded with the use of miRNAs. The authors could develop a classification system with a high accuracy based on miRNA expression profiles [40]. However, most of the miRNAs detected by Panarelli et al. did not play a role in the current analysis or in other studies [19, 29, 41]. It was shown that the miRNA expression profile is dependent on the tumor stage [19]. The inclusion criteria (two tumor samples of the same patient prior to and after the initiation of SSA treatment and no further systemic treatment) consecutively led to a numerically small patient collective. From a clinical point of view assessment of tumor tissue of the primary is impossible in most patients preoperatively since the majority of small intestine NETs cannot be reached endoscopically. Commonly these tumors are incidentally diagnosed by routine sonography of the liver. Thus, the initial tumor specimens are liver biopsies, subsequently leading to the initiation of SSA treatment. Therefore we had to analyze primary tumors as well as liver metastases.

Furthermore, previous studies have demonstrated that the miRNA expression profile differs between the primary tumor and (lymph node) metastases [19, 32], but the exact pathogenetic role of miRNAs remains ill-defined [22]. Previous studies have compared the primary tumor with adjacent normal tissue, liver metastases with normal liver or the primary tumor with liver metastases. The results of these heterogeneous analyses are inconsistent and concordant dysregulated miRNA signatures have not been published. Miller et al. demonstrated recently that the primary tumor and liver metastases even share 29 concordantly upregulated miRNAs [32]. Moreover, Heverhagen et al. demonstrated that liver metastases were nearly similar to the primary tumor [30]. The already characterized and published miRNAs (except let-7c), however, did not play a role in the present study. Regarding let-7c, our results are supported by Døssing et al. who recently have shown that SSA leads to an upregulation of let-7 family members [42], which inhibits the growth of carcinoid cell lines [31]. Therefore, the alterations seen in the present study may in part be induced by SSA treatment and adds further information in assessing the role of miRNAs in neuroendocrine tumors. Within the present study it was demonstrated for the first time that a biotherapy with SSAs leads to an alteration of the miRNA profile in tumor specimens of si-NETs. Three miRNAs were upregulated and 12 miRNAs were downregulated due to SSA treatment. Out of this grouped analysis two miRNAs (let-7c-5p and mir-3137) were found to be regulated concordantly in every single patient. Thus, it is suggested that the beneficial effects of somatostatin analogs are in part be mediated through these miRNAs. In silico analyses have revealed that the miRNAs, which are expressed differentially, have anti-tumor properties. Especially, the downregulation of let-7c-5p was associated with a worse outcome in pancreatic adenocarcinoma as well as in breast and lung cancer [43–45]. However, there are no reports available about the specific properties of let-7c-5p in GEP-NETs. Therefore, further prospective studies are demanded to elucidate the effect of let-7c-5p in GEP-NETs.

The alterations between the primary tumor and its metastases are of great interest and further studies are needed. However, analyzing the differences of primary tumors, its liver metastases and further distant metastases was beyond the scope of this manuscript. Further support for the relevance of the presented data, despite expected tumor heterogeneity, comes from the clinical observation that response to SSA treatment is homogenous between metastases and primary tumors. A solitary progression of distant metastases is only seen after years of treatment. Therefore, the results of this analysis with a homogenous patient collective consisting only of patients with si-NETs indicate that SSA treatment directly leads to alterations in the miRNA signature.

SSTR type-2 (SSTR2) is abundantly expressed on the surface of well-differentiated GEP-NETs [8, 42]. In this respect, in silico target gene analyses revealed that mir-185-3p directly interacts with SSTR2. Thus, the anti-tumor effect of SSAs might be in part mediated by downregulation of mir-185-3p. In this respect, it was shown recently that the downregulation of mir-185-3p is beneficial in nasopharyngeal carcinoma cell lines [46]. However, there is no distinct literature about specific effects of mir-3137, but target analyses showed that mir-3137 also interacts with SSTR2. Therefore, downregulation of mir-3137 might be a direct effect of the treatment with SSAs or the antitumor properties of SSAs are mediated at least in part via mir-3137 downregulation. The downregulation of mir-185-3p and mir-3137 and the upregulation of let-7c-5p or mir-215-5p may represent potential targets in the future. Thus, large prospective multicenter studies are necessary to address the future role of miRNAs in GEP-NETs.

Despite the promising results of the present study, there are several limitations as well. This is a retrospective analysis, however, based on a prospective led database. Furthermore, the source of the tumor samples is diverging. Due to the inclusion criteria (two tumor samples of each patient, no other systemic treatment) the patient cohort is numerically small and primary tumors as well as liver metastases had to be analyzed. In this respect, previous studies have demonstrated that the miRNA expression profile differs between the primary tumor and (lymph node) metastases [19, 32]. However, these miRNA signatures are not concordant and the previously published miRNAs did not play a role in the present manuscript, except of one miRNA. The SSA-treatment period is slightly different, which might influence miRNA expression profiles. However, the effect of any other systemic therapy could be ruled out since these patients were not included. In addition, from in vitro studies it is known that SSA inhibits cell growth within a short period of time and pharmacokinetic studies with humans revealed similar results [47, 48]. A control collective would be desirable. But, due to the rare incidence and the questioning a control collective was not provided.

## Conclusions

In conclusion, we demonstrat for the first time that the biotherapy with SSAs leads to an alteration of the miRNA expression profile. Especially, let-7c-5p and mir-3137 seem to be of great interest as these miRNAs were regulated uniformly in every patient. In this respect, future studies with larger cohorts are necessary to confirm the effects of miRNAs.

## Data Availability

The datasets used and/or analyzed during the current study available from the corresponding author on reasonable request.

## Abbreviations

CT: Cycle threshold
FFPE: Formalin-fixed paraffin-embedded
GEP-NET: Gastroenteropancreatic neuroendocrine tumor
miRNA: micro-RNA
NSCLC: Non-small cell lung cancer
si-NET: Neuroendocrine tumor of the small intestine
SSA: Somatostatin analog
SSTR: Somatostatin receptor
TLDA: Taqman Low Density Array

## References

[1] Maggard MA, O'Connell JB, Ko CY (2004). Updated population-based review of carcinoid tumors. Ann Surg.

[2] Yao JC, Hassan M, Phan A (2008). One hundred years after “carcinoid”: epidemiology of and prognostic factors for neuroendocrine tumors in 35,825 cases in the United States. J Clin Oncol.

[3] Bösch Florian, Bruewer Katharina, D'Anastasi Melvin, Ilhan Harun, Knoesel Thomas, Pratschke Sebastian, Thomas Michael, Rentsch Markus, Guba Markus, Werner Jens, Angele Martin K. (2018). Neuroendocrine tumors of the small intestine causing a desmoplastic reaction of the mesentery are a more aggressive cohort. Surgery.

[4] Saxena A, Chua TC, Sarkar A (2011). Progression and survival results after radical hepatic metastasectomy of indolent advanced neuroendocrine neoplasms (nens) supports an aggressive surgical approach. Surgery.

[5] Pavel M, Baudin E, Couvelard A (2012). Enets consensus guidelines for the management of patients with liver and other distant metastases from neuroendocrine neoplasms of foregut, midgut, hindgut, and unknown primary. Neuroendocrinology.

[6] Pavel M, O'Toole D, Costa F (2016). Enets consensus guidelines update for the management of distant metastatic disease of intestinal, pancreatic, bronchial neuroendocrine neoplasms (nen) and nen of unknown primary site. Neuroendocrinology.

[7] Auernhammer CJ, Spitzweg C, Angele MK (2018). Advanced neuroendocrine tumours of the small intestine and pancreas: clinical developments, controversies, and future strategies. Lancet Diabetes Endocrinol.

[8] Arnold R, Trautmann ME, Creutzfeldt W (1996). Somatostatin analogue octreotide and inhibition of tumour growth in metastatic endocrine gastroenteropancreatic tumours. Gut.

[9] Oberg KE, Reubi JC, Kwekkeboom DJ, Krenning EP (2010). Role of somatostatins in gastroenteropancreatic neuroendocrine tumor development and therapy. Gastroenterology.

[10] Caplin ME, Pavel M, Cwikla JB (2014). Lanreotide in metastatic enteropancreatic neuroendocrine tumors. N Engl J Med.

[11] Rinke A, Muller HH, Schade-Brittinger C (2009). Placebo-controlled, double-blind, prospective, randomized study on the effect of octreotide lar in the control of tumor growth in patients with metastatic neuroendocrine midgut tumors: a report from the promid study group. J Clin Oncol.

[12] Bartel DP (2004). Micrornas: genomics, biogenesis, mechanism, and function. Cell.

[13] He L, Hannon GJ (2004). Micrornas: small rnas with a big role in gene regulation. Nat Rev Genet.

[14] Heublein S, Albertsmeier M, Pfeifer D (2018). Association of differential mirna expression with hepatic vs. Peritoneal metastatic spread in colorectal cancer. BMC Cancer.

[15] Gao S, Zhao ZY, Wu R (2018). Prognostic value of micrornas in colorectal cancer: a meta-analysis. Cancer Manag Res.

[16] Li L, Sun Y, Feng M (2018). Clinical significance of blood-based mirnas as biomarkers of non-small cell lung cancer. Oncol Lett.

[17] Turashvili Gulisa, Lightbody Elizabeth D., Tyryshkin Kathrin, SenGupta Sandip K., Elliott Bruce E., Madarnas Yolanda, Ghaffari Abdi, Day Andrew, Nicol Christopher J. B. (2018). Novel prognostic and predictive microRNA targets for triple-negative breast cancer. The FASEB Journal.

[18] Fassan M, Baffa R (2013). Micrornas and targeted therapy: small molecules of unlimited potentials. Curr Opin Genet Dev.

[19] Li SC, Essaghir A, Martijn C (2013). Global microrna profiling of well-differentiated small intestinal neuroendocrine tumors. Mod Pathol.

[20] Roldo C, Missiaglia E, Hagan JP (2006). Microrna expression abnormalities in pancreatic endocrine and acinar tumors are associated with distinctive pathologic features and clinical behavior. J Clin Oncol.

[21] Ruebel K, Leontovich AA, Stilling GA (2010). Microrna expression in ileal carcinoid tumors: downregulation of microrna-133a with tumor progression. Mod Pathol.

[22] Malczewska A, Kidd M, Matar S (2018). A comprehensive assessment of the role of mirnas as biomarkers in gastroenteropancreatic neuroendocrine tumors. Neuroendocrinology.

[23] Zimmermann N, Knief J, Kacprowski T (2018). Microrna analysis of gastroenteropancreatic neuroendocrine tumors and metastases. Oncotarget.

[24] Bösch F, Hofmann K, Coenen M (2018). Surgical treatment of pnet – experience of a “high-volume” center. Surg Oncol.

[25] Abstracts: 136th congress of the german society of surgery (dgch). Innov Surg Sci. 2019;4(Suppl 1):s206–s307. 10.1515/iss-2019-2002. 10.1159/000498929 30884487

[26] Yin S, Bleul T, Zhu Y (2017). Mirnas are unlikely to be involved in retinoid receptor gene regulation in pancreatic cancer cells. Cell Physiol Biochem.

[27] Modlin IM, Oberg K, Chung DC (2008). Gastroenteropancreatic neuroendocrine tumours. Lancet Oncol.

[28] Nelson PT, Wang WX, Wilfred BR, Tang G (2008). Technical variables in high-throughput mirna expression profiling: much work remains to be done. Biochim Biophys Acta.

[29] Li SC, Khan M, Caplin M (2015). Somatostatin analogs treated small intestinal neuroendocrine tumor patients circulating micrornas. PLoS One.

[30] Heverhagen AE, Legrand N, Wagner V (2018). Overexpression of microrna mir-7-5p is a potential biomarker in neuroendocrine neoplasms of the small intestine. Neuroendocrinology.

[31] Dossing KB, Binderup T, Kaczkowski B (2014). Down-regulation of mir-129-5p and the let-7 family in neuroendocrine tumors and metastases leads to up-regulation of their targets egr1, g3bp1, hmga2 and bach1. Genes (Basel).

[32] Miller HC, Frampton AE, Malczewska A (2016). Micrornas associated with small bowel neuroendocrine tumours and their metastases. Endocr Relat Cancer.

[33] Bowden M, Zhou CW, Zhang S (2017). Profiling of metastatic small intestine neuroendocrine tumors reveals characteristic mirnas detectable in plasma. Oncotarget.

[34] Rizzo FM, Meyer T (2018). Liquid biopsies for neuroendocrine tumors: circulating tumor cells, DNA, and micrornas. Endocrinol Metab Clin N Am.

[35] Li S, Gao J, Gu J (2013). Microrna-215 inhibits relapse of colorectal cancer patients following radical surgery. Med Oncol.

[36] Vychytilova-Faltejskova P, Merhautova J, Machackova T (2017). Mir-215-5p is a tumor suppressor in colorectal cancer targeting egfr ligand epiregulin and its transcriptional inducer hoxb9. Oncogenesis.

[37] Ge G, Zhang W, Niu L (2016). Mir-215 functions as a tumor suppressor in epithelial ovarian cancer through regulation of the x-chromosome-linked inhibitor of apoptosis. Oncol Rep.

[38] Hou Y, Zhen J, Xu X (2015). Mir-215 functions as a tumor suppressor and directly targets zeb2 in human non-small cell lung cancer. Oncol Lett.

[39] Yao J, Zhang P, Li J, Xu W (2017). Microrna-215 acts as a tumor suppressor in breast cancer by targeting akt serine/threonine kinase 1. Oncol Lett.

[40] Panarelli N, Tyryshkin K, Wong JJM (2019). Evaluating gastroenteropancreatic neuroendocrine tumors through microrna sequencing. Endocr Relat Cancer.

[41] Mandal R, Hardin H, Baus R (2017). Analysis of mir-96 and mir-133a expression in gastrointestinal neuroendocrine neoplasms. Endocr Pathol.

[42] Døssing Kristina, Kjær Christina, Vikeså Jonas, Binderup Tina, Knigge Ulrich, Culler Michael, Kjær Andreas, Federspiel Birgitte, Friis-Hansen Lennart (2018). Somatostatin Analogue Treatment Primarily Induce miRNA Expression Changes and Up-Regulates Growth Inhibitory miR-7 and miR-148a in Neuroendocrine Cells. Genes.

[43] Fu X, Mao X, Wang Y (2017). Let-7c-5p inhibits cell proliferation and induces cell apoptosis by targeting ercc6 in breast cancer. Oncol Rep.

[44] Nwaeburu CC, Bauer N, Zhao Z (2016). Up-regulation of microrna let-7c by quercetin inhibits pancreatic cancer progression by activation of numbl. Oncotarget.

[45] Wu GQ, Chai KQ, Zhu XM (2016). Anti-cancer effects of curcumin on lung cancer through the inhibition of ezh2 and notch1. Oncotarget.

[46] Liu C, Li G, Ren S (2017). Mir-185-3p regulates the invasion and metastasis of nasopharyngeal carcinoma by targeting wnt2b in vitro. Oncol Lett.

[47] Buil-Bruna N, Garrido MJ, Dehez M (2016). Population pharmacokinetic analysis of lanreotide autogel/depot in the treatment of neuroendocrine tumors: pooled analysis of four clinical trials. Clin Pharmacokinet.

[48] Herrera-Martinez AD, Gahete MD, Pedraza-Arevalo S (2018). Clinical and functional implication of the components of somatostatin system in gastroenteropancreatic neuroendocrine tumors. Endocrine.

